# Identification of a Difluorinated Alkoxy Sulfonyl Chloride as a Novel Antitumor Agent for Hepatocellular Carcinoma through Activating Fumarate Hydratase Activity

**DOI:** 10.3390/ph16121705

**Published:** 2023-12-08

**Authors:** Jin Jin, Xujun Liang, Wu Bi, Ruijie Liu, Sai Zhang, Yi He, Qingming Xie, Shilei Liu, Ji-Chang Xiao, Pengfei Zhang

**Affiliations:** 1 NHC Key Laboratory of Cancer Proteomics, Department of Oncology, Xiangya Hospital, Central South University, Changsha 410008, China; JJ2054813945@163.com (J.J.); biwu0736@163.com (W.B.); summery365@163.com (R.L.); 218111125@csu.edu.cn (Q.X.); 228111137@csu.edu.cn (S.L.); 2 National Clinical Research Center for Geriatric Disorders, Xiangya Hospital, Central South University, Changsha 410008, China; 3 Key Laboratory of Organofluorine Chemistry, Shanghai Institute of Organic Chemistry, University of Chinese Academy of Sciences, Chinese Academy of Sciences, Shanghai 200032, China

**Keywords:** fenofibrate, hepatocellular carcinoma, MS-CETSA, fumarate hydratase

## Abstract

Fenofibrate is known as a lipid-lowering drug. Although previous studies have reported that fenofibrate exhibits potential antitumor activities, IC_50_ values of fenofibrate could be as high as 200 μM. Therefore, we investigated the antitumor activities of six synthesized fenofibrate derivatives. We discovered that one compound, SIOC-XJC-SF02, showed significant antiproliferative activity on human hepatocellular carcinoma (HCC) HCCLM3 cells and HepG2 cells (the IC_50_ values were 4.011 μM and 10.908 μM, respectively). We also found this compound could inhibit the migration of human HCC cells. Transmission electron microscope and flow cytometry assays demonstrated that this compound could induce apoptosis of human HCC cells. The potential binding sites of this compound acting on human HCC cells were identified by mass spectrometry-cellular thermal shift assay (MS-CETSA). Molecular docking, Western blot, and enzyme activity assay-validated binding sites in human HCC cells. The results showed that fumarate hydratase may be a potential binding site of this compound, exerting antitumor effects. A xenograft model in nude mice demonstrated the anti-liver cancer activity and the mechanism of action of this compound. These findings indicated that the antitumor effect of this compound may act via activating fumarate hydratase, and this compound may be a promising antitumor candidate for further investigation.

## 1. Introduction

Primary liver cancer was the leading cause of cancer-related deaths worldwide in 2020 and has the sixth highest incidence of malignancy (8.3%), with approximately 906,000 new cases and 830,000 deaths [1]. Hepatocellular carcinoma (HCC) comprises approximately 85% of the total cases of primary liver cancer worldwide [2]. HCC severely threatens the life and health of human beings and brings a serious burden to families and society. HCC most often occurs in patients with a history of cirrhosis due to long-term alcoholism, non-alcoholic fatty liver disease, or hepatitis C virus (HCV) infection. The continuous cycle of inflammation and healing in liver cells is believed to be the root cause of the development of HCC. Numerous therapeutic measures have been developed, such as surgical resection, chemotherapy, radiotherapy, and liver transplantation [3]; however, the coexistence of inflammation and cirrhosis complicates the early diagnosis of HCC [4]. Surgical resection is the cornerstone of early-stage HCC. Detection of early-stage HCC means that the cancer can be treated with surgical resection, gaining a relatively favorable prognosis with a 5-year survival rate of more than 70% [5]. In addition, the recurrence rate after resection is about 80%, with a high fatality rate [6]. Furthermore, the majority of HCC patients are already in the middle and late stages when diagnosed, losing the chance to receive surgery, and can only choose non-surgical resection. Chemotherapy, especially molecular targeted drug therapy, is attracting increasing attention in the treatment of middle and late-stage patients with HCC. In 2016, the only first-line systemic therapy for unresectable HCC approved by the Food and Drug Administration was sorafenib, which is a multi-targeted oral small-molecule tyrosine kinase inhibitor (TKI) that can curb cell proliferation of the tumor cell via inhibiting the activity of Raf-1, B-Raf, and kinases in the Ras/Raf/MEK/ERK signaling pathway. Moreover, sorafenib can inhibit angiogenesis by targeting the receptor tyrosine kinase (c-Kit), FMS-like tyrosine kinase (FLT-3), vascular endothelial growth factor receptor (VEGFR)-2, VEGFR-3, platelet-derived growth factor receptor (PDGFR-β), and other tyrosine kinases [7,8,9]. Unfortunately, most patients do not experience a long-term benefit, mainly due to the early onset of sorafenib resistance [10]. Compared with other solid cancers, HCC is considered a chemotherapy-resistant tumor, with excessive expression of dihydropyrimidine dehydrogenase, the P-glycoprotein gene product, and the multidrug resistance gene, MDR-1, so systemic chemotherapy is not routinely used [11,12,13]. The prognosis of HCC is disappointing, with a 3-year survival rate of 12.7% and a median survival of 9 months [14]. 

Fenofibrate is regarded as a third-generation fibrate lipid-lowering drug that has been suitable for patients with severe hypertriglyceridemia and mixed dyslipidemia for many years in clinical practice. Its ester bond is rapidly hydrolyzed in vivo by esterases in tissues and plasma to form the active metabolite fenofibrate acid acting as a hypolipidemic agent by activating peroxisome proliferator-activated receptor alpha (PPRAα) [15,16,17]. In recent years, fenofibrate has been found to exert antitumor effects by inhibiting tumor cell proliferation and metabolism, inhibiting neovascularization, inhibiting cell motility migration, inducing apoptosis, and blocking the cell cycle. It is not dependent on the PPRAα pathway [18,19,20,21,22], and many studies have proven that fenofibrate can be used as an important potential antitumor agent, demonstrated by a variety of human malignant tumor types, such as pancreatic cancer [18], breast cancer [23], hepatocellular carcinoma [19,24], high-grade glioma [17,25,26,27], prostate cancer [20,28,29], endometrial cancer [30,31], oral cancer [32,33], lung cancer [34,35], lymphoma and multiple myeloma [36], mantle cell lymphoma [37], neuroblastoma [38], melanoma [39]. Fenofibrate may also increase the efficacy of vaccines against solid tumors by reprogramming cells within tumors to increase fatty acid metabolism [40]. However, the inhibition effect of fenofibrate on tumor cells in vitro and in vivo is less pronounced, and its half-inhibitory concentration (IC_50_) values for tumor cell growth inhibition are relatively large, reaching up to 200 μM [41,42].

There are many known fibric acid derivatives, but fenofibrate was most studied in antitumor research as described above, and the inhibition effect of fenofibrate on tumor cells is unsatisfactory. The pharmacokinetics and safety of fenofibrate are well known in clinical practice; therefore, a series of fenofibrate derivatives based on the chemical structure formula of fenofibrate have been designed to improve the outcome of the treatment of HCC. The present study investigated the anticancer potency of the screened fenofibrate derivative SIOC-XJC-SF02, 2-{4-[4-(2-Fluorosulfonyl-vinyl)-benzoyl]-phenoxy}-2-methyl-propionic acid isopropyl ester (C22H23FO6S) (The spectral results for SIOC-XJC-SF02 are presented in the Supplementary Material.) in HCC, in vitro and in vivo. The underlying mechanisms were also investigated. Our data indicated that compound SIOC-XJC-SF02 inhibited cell proliferation and induced cell apoptosis via the targeting of fumarate hydratase. This study suggests that fenofibrate derivative SIOC-XJC-SF02 is a promising leading compound for anti-hepatocellular carcinoma.

## 2. Results

### 2.1. Compound SIOC-XJC-SF02 Decreases the Viability of Human HCC Cells

**Figure 1:**
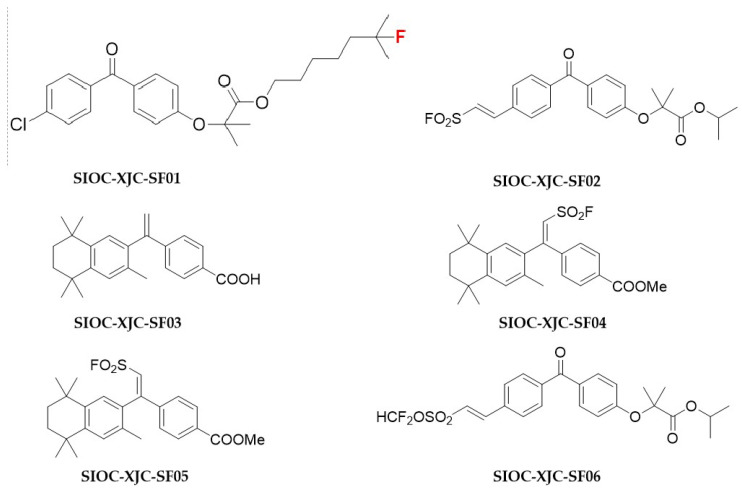
Chemical structure of the fenofibrate derivatives.

**Figure 2:**
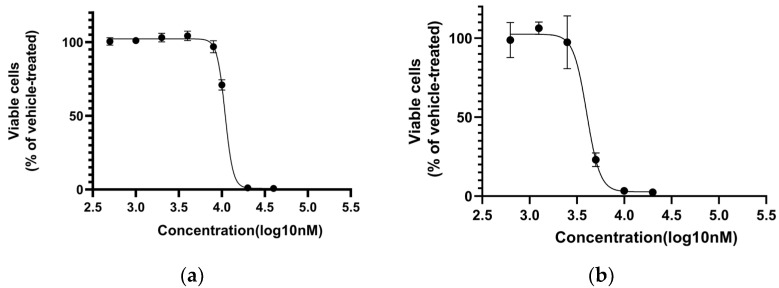
Influence of human hepatocellular carcinoma cells on proliferation induced by compound SIOC-XJC-SF02. (**a**,**b**) show the antitumor activity of SIOC-XJC-SF02 on HepG2 cells and HCCLM3 cells, respectively (IC_50_: 10.908 μM and 4.011 μM, respectively).

To identify the antitumor properties of novel fenofibrate derivatives, six compounds were prepared to evaluate their antitumor activities (Figure 1 and Table 1). The fenofibrate derivative SIOC-XJC-SF02 (Figure 2) showed potent antitumor activity compared with all of the other compounds, and the IC_50_ values of compound SIOC-XJC-SF02 acting on HepG2 cells were 10.908 μM and 4.011 μM against HCCLM3 cells. The effects of compound SIOC-XJC-SF06 acting on HepG2 cells were also evaluated; IC_50_ values were 4.182 μM, but the amount of compound SIOC-XJC-SF06 synthesis was insufficient. These results indicated that the cell viability of HepG2 cells and HCCLM3 cells incubated with increasing concentrations of compound SIOC-XJC-SF02 decreased in a dose-dependent manner. Furthermore, it was observed that compound SIOC-XJC-SF02 exhibited better antitumor activity on HCCLM3 cells compared with HepG2 cells.

Primary analysis of the structure-activity relationship of these fenofibrate derivatives indicated that the Cl atom of fenofibrate replaced by different chemical atomic groups was likely significant for their anti-liver cancer activity, such as SIOC-XJC-SF02 and SIOC-XJC-SF06.

### 2.2. Compound SIOC-XJC-SF02 Induces Apoptosis of Human HCC Cells

**Figure 3:**
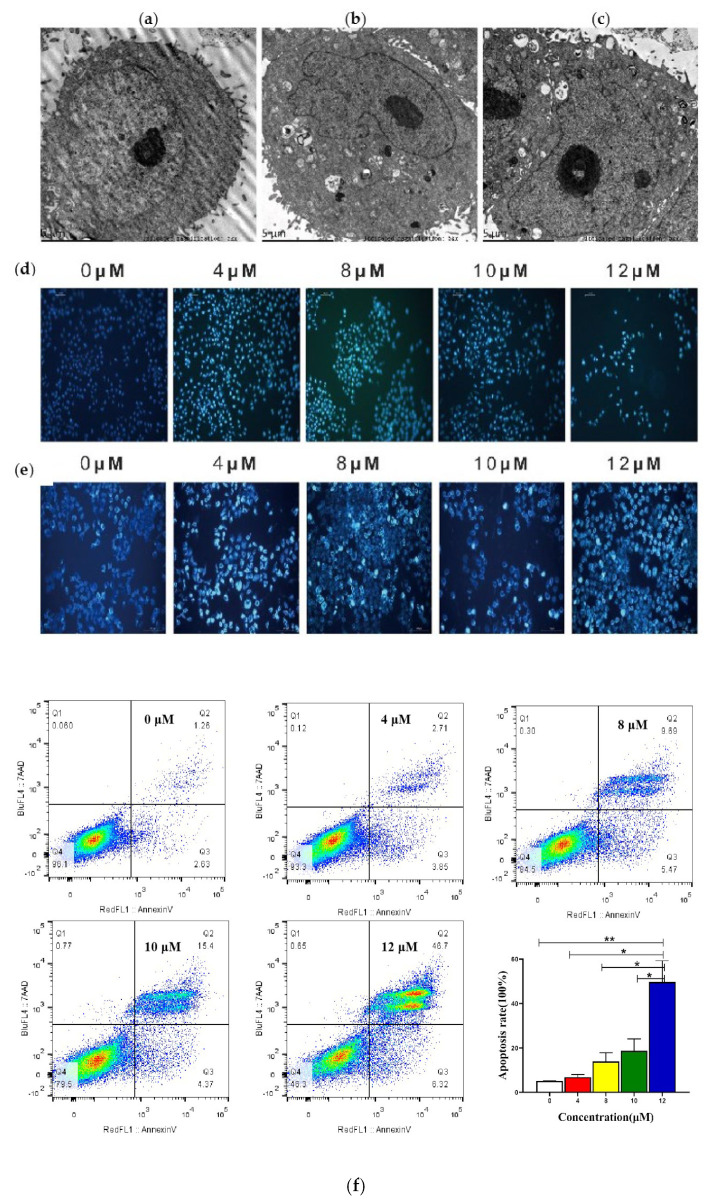

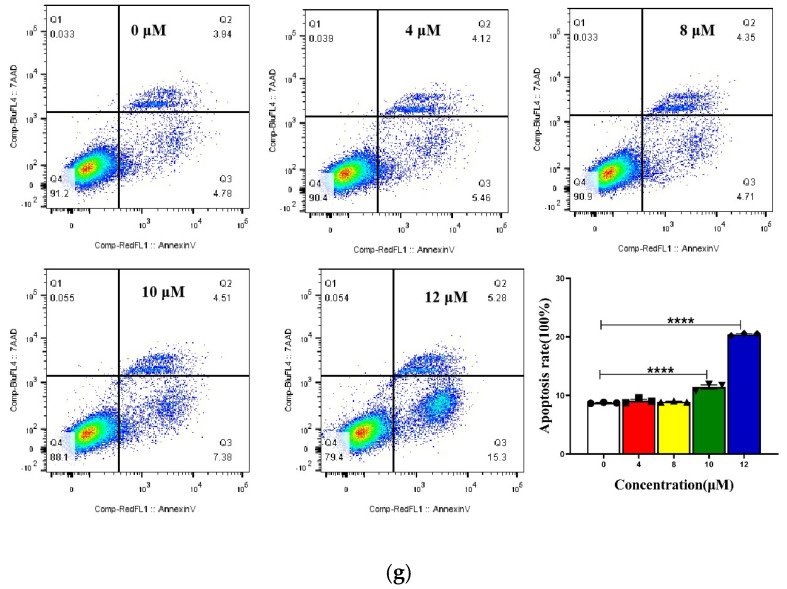
Changes of human hepatocellular carcinoma cells induced by compound SIOC-XJC-SF02. (**a**–**c**) Compound SIOC-XJC-SF02 (0–8 μM) induced morphological changes in HepG2 cells after 24 h (5000×, bar = 5 μm). (**d**,**e**) Compound SIOC-XJC-SF02 (0–12 μM) induced morphological changes in HepG2 cells (**d**) and HCCLM3 cells (**e**) after 48 h. (**f**,**g**) Compound SIOC-XJC-SF02 (0–12 μM) induced morphological changes in HepG2 cells (**f**) and HCCLM3 cells (**g**) after 48 h (Four-quadrant diagrams: Abscissa: RedFL1::AnnexinV, Ordinate: BluFL4::7AAD.). All experimental data was analyzed using GraphPad software (https://www.graphpad.com/) and ordinary one-way ANOVA. * *p* < 0.05, ** *p* < 0.01, **** *p* < 0.0001 vs. control.

To determine the effect of compound SIOC-XJC-SF02 on cell apoptosis, transmission electron microscopy assay and Hoechst 33258 staining were conducted to observe the morphological changes of apoptotic cells, and flow cytometry was performed to evaluate the cell apoptotic ratio. The results of the transmission electron microscopy assay and Hoechst 33258 staining indicated that HepG2 cells and HCCLM3 cells treated with compound SIOC-XJC-SF02 showed typical apoptotic morphological changes compared with the vehicle. In cells treated with SIOC-XJC-SF02, it could be observed that chromatin edged at the periphery of the nuclear membrane, chromatin condensed, and cytoplasm was concentrated (Figure 3a–e). 

Flow cytometry analysis was performed to detect the apoptosis of HepG2 cells and HCCLM3 treated with different concentrations of SIOC-XJC-SF02 for 48 h. As shown in Figure 3f,g, the apoptosis rate increased significantly with the rising concentration of compound SIOC-XJC-SF02.

### 2.3. Compound SIOC-XJC-SF02 Inhibits Migration of Human HCC Cells

The wound healing assay and transwell assay are vital for assessing the migration ability of tumor cells. Therefore, the two methods mentioned above were used to indicate the anti-migration effects of compound SIOC-XJC-SF02 on human HCC cells. 

**Figure 4:**
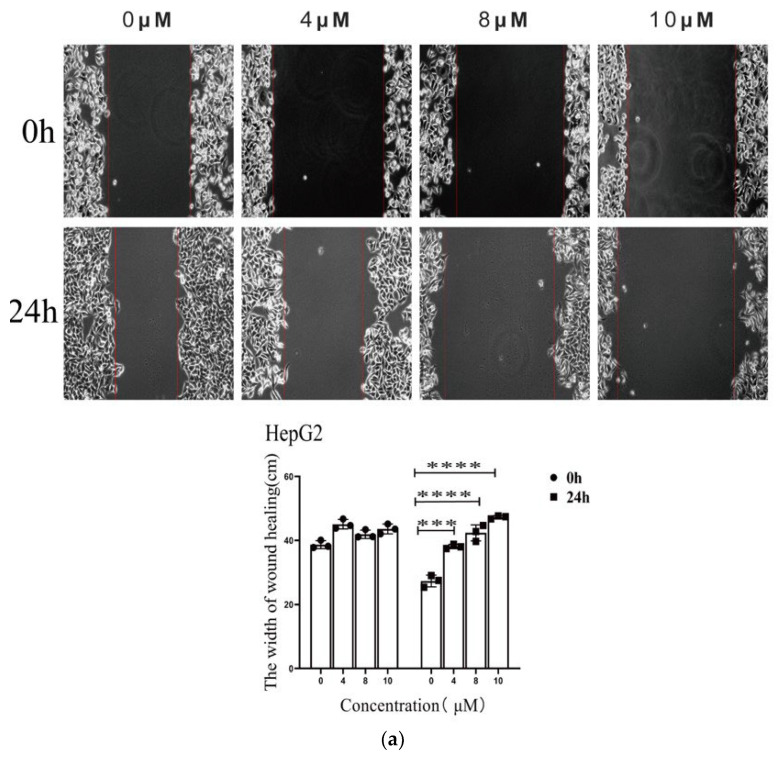

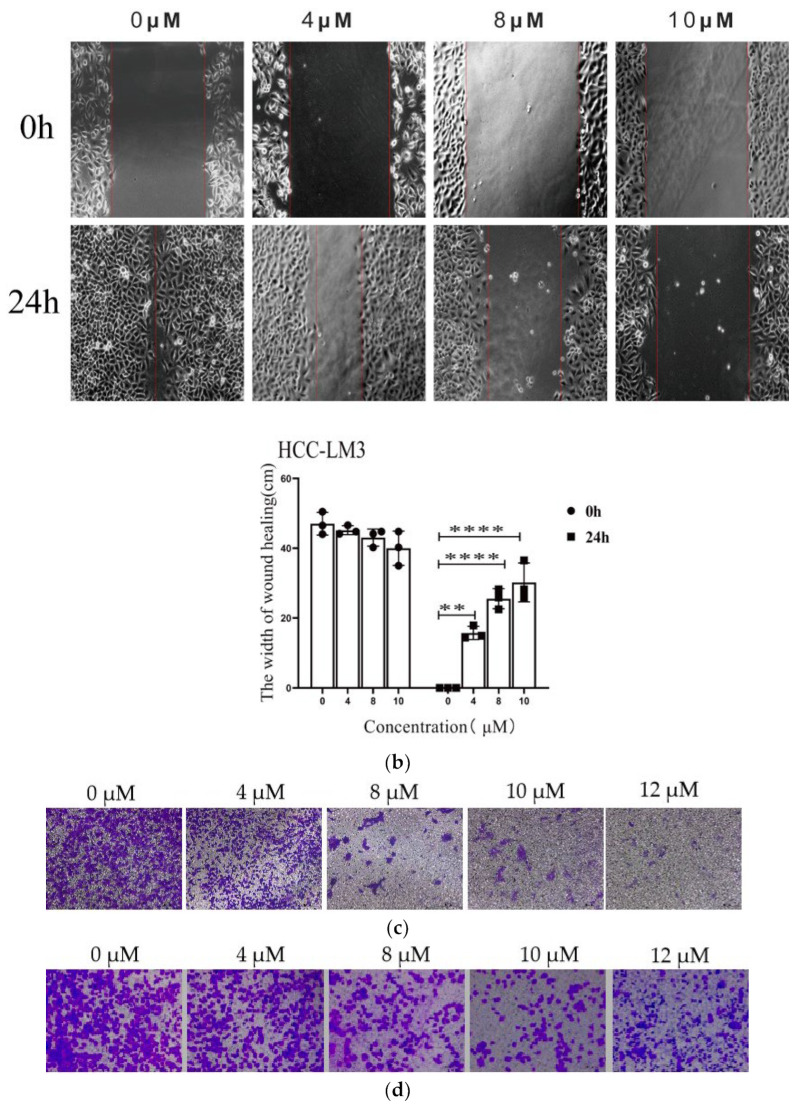
Influence of SIOC-XJC-SF02 on migration in HepG2 cells and HCCLM3 cells. HepG2 cells (**a**) and HCCLM3 cells (**b**) were treated with SIOC-XJC-SF02 (0, 4, 8, and 10 μM) to observe the scratch distance after grown for 24 h. HepG2 cells (**c**) and HCCLM3 cells (**d**) were treated with SIOC-XJC-SF02 (0, 4, 8, 10, and 12 μM) for 24 h to observe the number of migratory cells. The data of the scratch experiments were analyzed using GraphPad software and ordinary one-way ANOVA. ** *p* < 0.01, *** *p* < 0.001, and **** *p* < 0.0001 vs. control.

As shown in Figure 4, the exposure of HepG2 cells and HCCLM3 cells to compound SIOC-XJC-SF02 resulted in significant suppression of migration. Compared with the control group, cells treated with SIOC-XJC-SF02 formed a wider scratch distance and decreased in a concentration-dependent manner. HepG2 cells incubated with 10 μM compound SIOC-XJC-SF02 showed a significant scratch distance (Figure 4a), and the migration was completely suppressed with 12 μM of SIOC-XJC-SF02 (Figure 4c).

### 2.4. Preliminary Exploration and Identification of Potential Binding Sites of Compound SIOC-XJC-SF02 against HepG2 Cells

**Figure 5:**
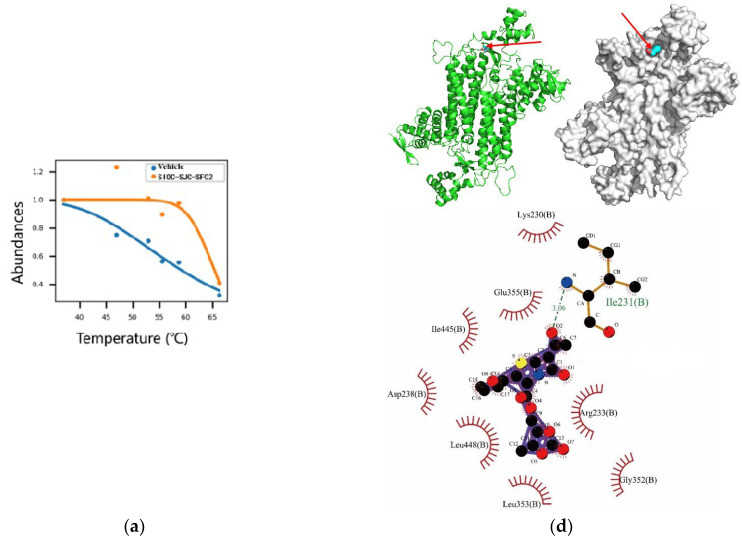

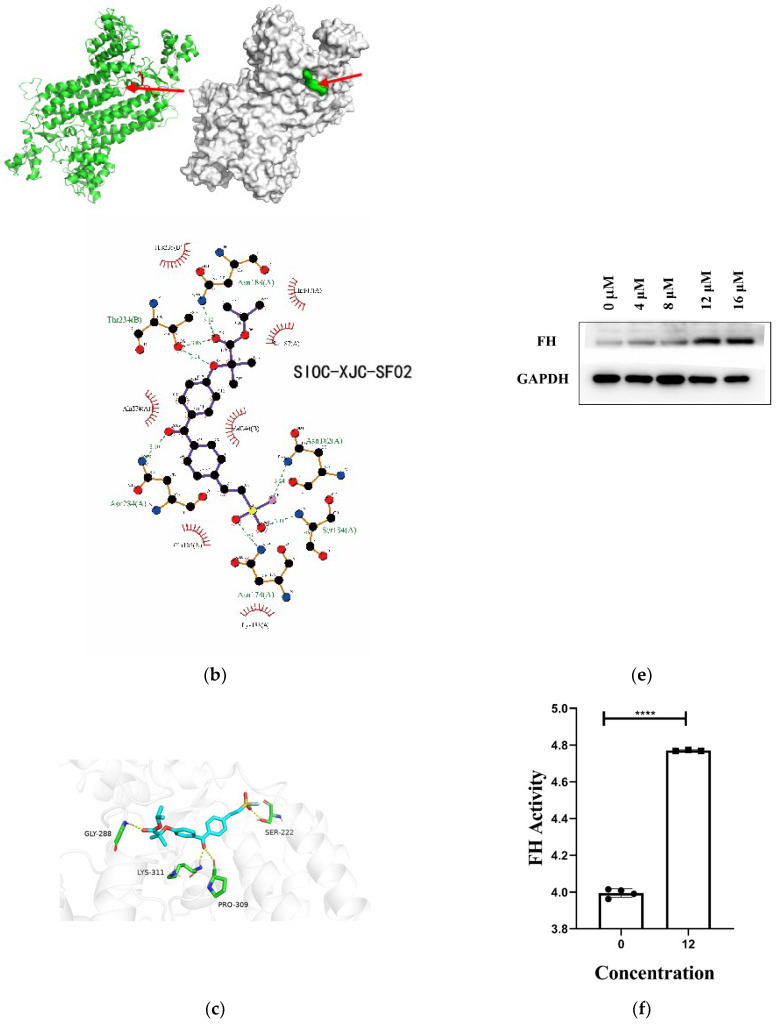
Compound SIOC-XJC-SF02 (0–16 μM) changed the expression level of fumarate hydratase (FH). (**a**) The melting curve of fumarate hydratase was obtained via MS-CETSA. (**b**) Results of molecular docking of compound SIOC-XJC-SF02 with fumarate hydratase. Red arrows indicate compound SIOC-XJC-SF02. (**c**) Specific locations of the docking of compound SIOC-XJC-SF02 combined with fumarate hydratase. (**d**) Results of molecular docking of compound fumarate hydratase-IN-1 with fumarate hydratase. Red arrows indicate compound fumarate hydratase-IN-1. (**e**) The expression level of FH was determined by Western blotting using specific antibodies. GAPDH was used as an internal control. (**f**) The expression level of mitochondrial fumarate hydratase activity was significantly decreased with the treatment of 12 μM SIOC-XJC-SF02 in HCCLM3 cells. These data were analyzed by GraphPad software and ordinary one-way ANOVA. **** *p* < 0.0001, compared to the control.

To further explore the binding sites of compound SIOC-XJC-SF02, mass spectrometry-cellular thermal shift assay (MS-CETSA) was conducted by exploiting the biophysical properties of proteins and their stability when combined with the compound [43]. The results showed that the score of the melting curve fitting for fumarate hydratase (A0A0S2Z4C3) was 1.035 (Figure 5a). We speculated that the binding sites of compound SIOC-XJC-SF02 against hepatocellular carcinoma cells may be fumarate hydratase (A0A0S2Z4C3).

The results of the molecular docking assay showed that compound SIOC-XJC-SF02 may combine with fumarate hydratase (Figure 5b), and the affinity was −7.4 (kcal/mol), and the location of compound SIOC-XJC-SF02 combined with fumarate hydratase were four hydrogen bonds: glycine, lysine, proline, and serine (Figure 5c). Fumarate hydratase-IN-1, as the control, was chosen to perform molecular docking for fumarate hydratase, and the results are shown in Figure 5d. The molecular docking binding energy of fumarate hydratase-in-1 was −8.4 (kcal/mol), and the result was stable. Moreover, its binding energy was similar to SIOC-XJC-SF02.

As shown in Figure 5e,f, the mitochondrial FH activity was affected by compound SIOC-XJC-SF02 in a dose-dependent manner, and Western blot results further indicated that compound SIOC-XJC-SF02 enhanced the activity of FH.

### 2.5. Compound SIOC-XJC-SF02 Suppresses Tumor Growth of HepG2 Cell Xenografts in Nude Mice

**Figure 6:**
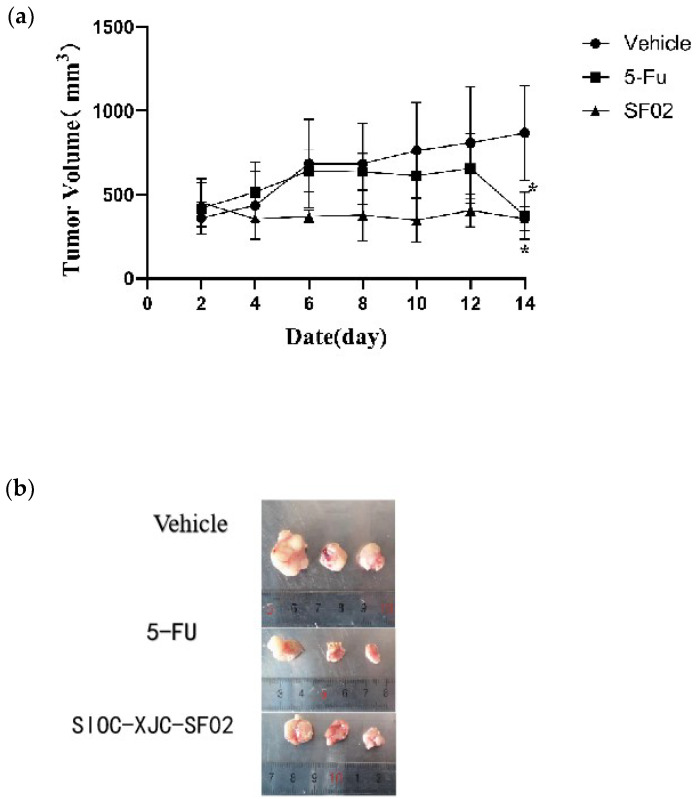
Antitumor effects of compound SIOC-XJC-SF02 against xenografted tumors in nude mouse. (**a**) Tumor volumes of the animals were measured every two days. (**b**) Photographed tumors from corresponding animals. Experimental data were analyzed by using GraphPad software and ordinary one-way ANOVA. * *p* < 0.05, compared to the vehicle.

To evaluate the effect of compound SIOC-XJC-SF02 on the growth and development of liver cancer in vivo, HepG2 cell xenografts in nude mice were established. The compound SIOC-XJC-SF02 group (20 mg/kg), 5-FU as a positive control group (30 mg/kg), and the vehicle as the control group were administered by intraperitoneal injection every two days. As shown in Figure 6, compared to the vehicle group, tumor volume was significantly decreased with the treatment of compound SIOC-XJC-SF02, and the antitumor effect was approximately the same as the 5-FU group on the 14th day (Figure 6a).

**Figure 7:**
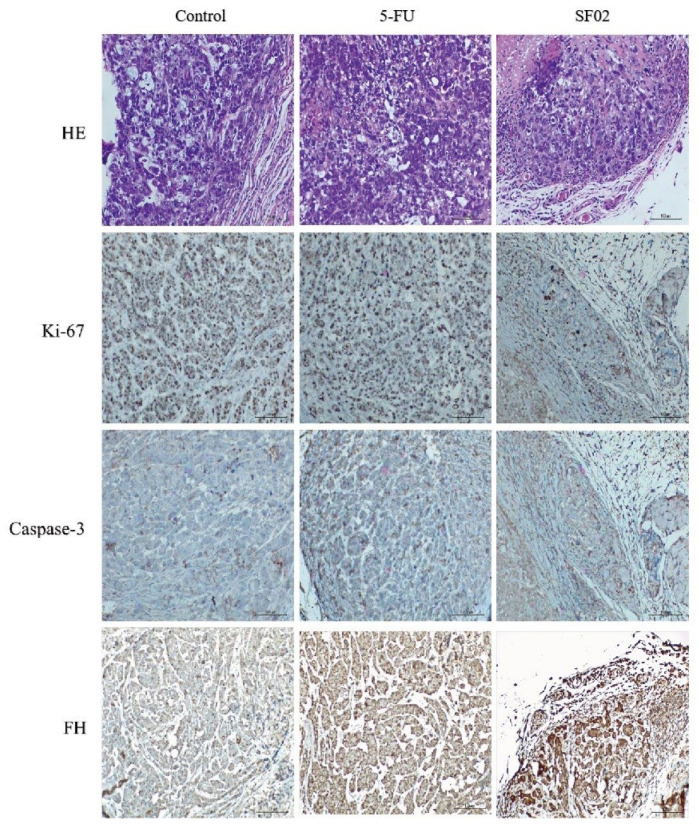
Representative images of immunohistochemical analysis for Ki-67, caspase-3, and fumarate hydratase in tumor sections (magnification: 400×, Scale bar: 4 μm).

Immunohistochemical analysis was performed to detect the expression levels of Ki-67, caspase-3, and fumarate hydratase in tumor tissues (Figure 7). Ki-67, which represents a marker of cell proliferation, showed a significant decrease in 5-FU and SIOC-XJC-SF02 groups. In addition, the expression levels of caspase-3 and fumarate hydratase were increased in the 5-FU and SIOC-XJC-SF02 groups. These results were consistent with the conclusions of in vitro experiments.

## 3. Discussion

This manuscript investigated six fenofibrate derivatives for their antitumor effects, and a difluorinated alkoxy sulfonyl chloride (compound SIOC-XJC-SF02) performed promising antitumor effects, which has not been reported previously. The ADMET properties of SIOC-XJC-SF02 predicated via SwissADME (http://www.swissadme.ch/index.php, accessed on 26 October 2023) are shown as follows: pharmacokinetics—GI absorption, High; BBB permeant, No; P-gp substrate, No; Log *k*_p_ (skin permeation) −5.58 cm/s; bioavailability score 0.55; lipophilicity, Log *P*_o/w_ (MLOGP) 2.55. Furthermore, the effects on anti-liver cancer and underlying mechanisms of compound SIOC-XJC-SF02 were revealed in cell and animal experiments. 

Compound SIOC-XJC-SF02 showed better antitumor activities on human HCC cells compared with other fenofibrate derivatives. The primary analysis of the structure–activity relationship stated that the Cl atom of fenofibrate replaced by different chemical atomic groups is significant for the antitumor effect of fenofibrate derivatives. The IC_50_ value of HepG2 cells treated with compound SIOC-XJC-SF02 differed from compound SIOC-XJC-SF06, considering that the Cl atom of fenofibrate was replaced by different chemical atomic groups. Therefore, more studies are needed to research the differences in the future to optimize the chemical structure of fenofibrate derivatives.

Induction of cell apoptosis is a momentous objective of antitumor therapy [44]. In this manuscript, we observed that under the treatment of compound SIOC-XJC-SF02, the morphological changes of HepG2 cells and HCCLM3 cells occurred. Therefore, we estimated whether the treatment of compound SIOC-XJC-SF02 induced cancer cell apoptosis. The results showed that compound SIOC-XJC-SF02 increased the apoptosis rate significantly in a dose-dependent manner in HCC cells. These results identified that compound SIOC-XJC-SF02 exerted an antitumor effect via inducing apoptosis in human HCC cells.

The inhibition effect of compound SIOC-XJC-SF02 against human HCC cells was approximately the same as the positive control 5-FU on the 14th day in vivo. However, the tumor volumes of nude mice in the compound SIOC-XJC-SF02 group were almost always lower than in the 5-FU group. These results require further study for the optimization of the chemical structure of compound SIOC-XJC-SF02 to produce a low-toxicity and effective anti-liver cancer drug in the future.

Identification of binding sites of drugs is a crucial step for an approved drug [45]. The compound SIOC-XJC-SF02 cannot be modified with present experimental techniques. Therefore, MS-CETSA was selected to discover and identify the potential binding sites of compound SIOC-XJC-SF02 after consulting many relevant literature [43,46]. MS-CETSA is based on the biophysical principle of ligand-induced thermal stabilization of proteins, meaning when the proteins bind to a drug molecule, the melting temperature of the proteins increases, resulting in the detection of proteins in the soluble fraction [43]. Theoretically, any modification of protein will affect its thermal stability [47]. Compounds that alter the melting point of proteins are considered as the binding agents for the proteins. MS-CETSA was used to report that P2X4 is a cellular target protein of indophagolin [48]. Compound DD100097 was demonstrated to act on LdNMT(N-myristoyltransferase, NMT) in the Leishmania protozoa parasite [49]. Therefore, MS-CETSA was performed to explore the binding sites of compound SIOC-XJC-SF02 acting on the HCC cells. The results indicated that mitochondrial fumarate hydratase may be the antitumor binding site of compound SIOC-XJC-SF02 acting on human HCC cells. The above results were further supported by mitochondrial fumarate hydratase activity assay, molecular docking assay, and Western blot assay.

The mitochondrial fumarate hydratase acts as a homotetramer. The gene of FH is located at 1q43, consists of 22,153 base pairs, transcribed into 10 exons, and then translated into 510 amino acid monomers (NCBI gene ID 2271, UniProt P07954). Each monomer has three structural domains: the central structural domain responsible for the interaction between the monomers, the N-terminal cleavage enzyme 1 structural domain, and the C-terminal fumarase C structural domain [50]. FH is a key enzyme in the tricarboxylic acid cycle (also called the citric acid cycle), responsible for the generation of cellular energy through oxidative phosphorylation, which reversibly catalyzes water reaction to convert fumarate to L-malate [51,52]. The absence of FH and subsequent accumulation of fumarate lead to epithelial-to-mesenchymal transition (EMT), a phenotypic transition associated with the initiation, invasion, and metastasis of cancer [53]. Fumarate inhibits HPHs belonging to the 2-oxoglutarate-dependent dioxygenase family. Other members of this family are the enzymes of 10–11 translocation (TET) and are involved in DNA methylation. TET enzymes oxidize 5-methylcytosine to 5-hydroxymethylcytosine (5-HMC) and other oxidized methylcytosines, ultimately allowing DNA demethylation [54]. TET2 mutations with importance in epigenetic regulation were identified in several human cancers [55]. Fumarate is identified as an inhibitor of TETs, and demonstrated that fumarate downregulated 5-HMC levels, introducing a potential epigenetic role for fumarate [54]. In a subsequent study, fumarate’s role is associated with EMT epigenetic regulation [53]. Normally, mitochondria provide most of the energy needed to sustain cell life activities. However, when it is necessary to eliminate cancer cells physiologically or therapeutically, mitochondria can trigger apoptosis and cell-programmed death [56]. It has been widely reported that FH is closely associated with the development of various tumors, such as uterine smooth muscle tumors [57], renal cell carcinoma [58], pancreatic cancer [59], gastric cancer [60], and lung cancer [61]. Human type 2 papillary renal cell carcinoma (PRCC2) cells were cultivated, and the results showed that accumulation of fumarate in cells lacking FH contributed to PTEN inhibition to activate PI3K/AKT signaling, tumor growth in PRCC2, and the development of sunitinib resistance in PRCC2 cells [58]. FH has a protective effect in maintaining appropriate macrophage cytokine and interferon responses [62]. However, there are not so many reports on the relationship between the enhanced activity of fumarate hydratase and tumor growth. Compound SIOC-XJC-SF02, a novel derivative of fenofibrate, may exert its antitumor effects indirectly through other pathways to enhance the activity of fumarate hydratase or directly in combination with it.

## 4. Materials and Methods

### 4.1. Materials and Reagent

Six fenofibrate derivatives were synthesized at the Shanghai Institute of Organic Chemistry (purity: >98%, HPLC) according to a previous protocol [63]. The primary antibodies, including Ki-67 and Caspase-3, and the second antibodies used in this study were purchased from Cell Signaling Technology (CST, Danvers, MA, USA). The primary antibody, fumarate hydratase, was purchased from the Proteintech Group (Wuhan, China).

### 4.2. Cell Lines and Cell Culture

Human hepatoma carcinoma cell lines HepG2 and HCCLM3 were provided by the Cancer Research Institute of Central South University (Changsha, China). HepG2 cells were cultured in DMEM (high glucose) (Gibco, Waltham, MA, USA) supplemented with 10% (*v*/*v*) fetal bovine serum (FBS, Gibco, USA) containing 1% penicillin/streptomycin (Gibco, USA). HCCLM3 cells were cultured in RMPI-1640 medium (FBS, Gibco, USA) with 10% fetal bovine serum containing 1% penicillin/streptomycin. These cell lines were cultured at 37 °C in a humidified 5% CO_2_ incubator.

### 4.3. Cell Proliferation Assay

The Cell Counting Kit-8 (Beyotime, Shanghai, China) was used to evaluate cell proliferation, which was performed according to the manufacturer’s instructions. Cells ((1–3) × 10^3^ per well) were seeded in a 96-well plate (Corning, New York, NY, USA) in 100 μL complete medium for 8–24 h, added with different concentrations of test compounds. The compounds were solubilized in DMSO (<0.1% in the final concentration). After treatment for 48 h, 10 μL of the CCK-8 reagent was added with 100 μL of pure DMEM (high glucose) or RMPI-1640 medium and incubated with cells for 2 h. The optical density was determined at 450 nm. IC_50_ values were determined through the dose–response curves.

### 4.4. Transmission Electron Microscope Assay

Cells for transmission electron microscope assay were harvested by a cell scraper after compound treatment, centrifuged, fixed with 2.5% glutaraldehyde, washed with PBS, dehydrated, permeabilized, embedded, sectioned, stained, finally observed by transmission electron microscope.

### 4.5. Transwell Assay

Cells (2 × 10^3^ per well) were seeded in the top chamber of an 8.0 µm membrane of a 24-well plate, which were inoculated with 100 µL DMEM. Different concentrations of compound with 500 μL DMEM containing 10% FBS were added in the bottom chamber. After treatment for 24 h, the cells were fixed with 4% paraformaldehyde, stained with 0.1% crystalline violet, and photographed.

### 4.6. Wound Healing Assay

The 6-well plate was used for the wound healing assay, the bottom of which was drawn horizontal lines; 5 × 10^5^ cells per well were seeded and filled the whole well, and the same sterile 200 µL pipette tip was used to scratch along the direction vertically to the marker line, incubated with different concentrations of compound, and photographed after 0 h and 24 h.

### 4.7. Hoechst 33258 Staining

Cells (2 × 10^5^/well) were seeded in the 6-well plate, grown for 24 h, and co-incubated with different concentrations of the compound for 48 h. Cells were washed three times with PBS, fixed with 4% paraformaldehyde (*v*/*v*) for 5 min at room temperature, and then washed with PBS, stained with Hoechst 33258 solution (Solarbio, Beijing, China) at a final concentration of 1 mg/mL for 10 min at room temperature, finally subjected to UV microscopy immediately with filters for blue fluorescence (Leica, Wetzlar, Germany). 

### 4.8. Annexin V/PI Flow Cytometry Assay for Apoptosis

Cells (3 × 10^5^ cells per well) were seeded in 6-well plates, grown for 24 h, treated with different concentrations of compound for 48 h, collected by trypsinization, washed twice with cold PBS by centrifugation for 5 min at 1500× *g*, then, the cells were labeled with Annexin V-FITC and PI (Proteintech, Rosemont, IL, USA) for 30 min in darkness at room temperature, as described by the instruction of products, and then subjected to FACScan flow cytometer for analysis. Finally, the experimental data were analyzed using the FlowJo VX software.

### 4.9. Protein Extraction and Western Blot Analysis

Cells treated with different concentrations of compound SIOC-XJC-SF02 were harvested in ice-cold whole-cell extract buffer RIPA supplemented with PMSF (Beyotime Biotech, Shanghai, China). The collected lysates were centrifuged at 14,000× *g* for 15 min at 4 °C, and the protein concentration was measured via the BCA Protein Assay Kit (CWBiotech, Beijing, China) according to the manufacturer’s instructions. Proteins were separated by 10% SDS-PAGE, transferred to polyvinylidene difluoride membranes (PVDF, Millipore, Burlington, MA, USA), and then membranes were blocked with 5% fat-free milk in 1× TBS with 0.1% Tween-20 (Bio Froxx, Einhausen, Germany) for 2 h at room temperature. The membranes were incubated with primary antibodies overnight at 4 °C, then washed with PBS added with Tween-20 for 30 min and incubated with secondary antibodies for 2 h at room temperature. The ECL Kit (Vazyme, Nanjing, China) visualized protein bands on the membrane, and chemiluminescent images were collected by the Syngene G:BOX Chemi XX9 imager (Syngene, Frederick, MD, USA).

### 4.10. In Vivo Animal Experiment

BALB/c male nude mice (4–5 weeks old) were purchased from BEIJING HFK Biot SCIENCE CO., Ltd. (Beijing, China) and raised in individually ventilated cages in a specific pathogen-free environment in the Laboratory Animal Center of Central South University. To build the xenografted tumor model, HepG2 cells (8 × 10^6^ cells per mouse) were inoculated subcutaneously into the left armpit of nude mice after being sterilized with 75% alcohol. When the tumor volume reached ~50 mm^3^, nude mice were randomly divided into three groups (n = 5/group): vehicle control (experimental solvent with compound-free) (group 1); 5-FU, 30.0 mg/kg every 2 days (group 2); compound SIOC-XJC-SF02, 20.0 mg/kg every 2 days (group 3). The compound was dissolved in PEG300 (Selleck, Texas, USA) and Tween-80 (Bio Froxx, Einhausen, Germany). Nude mice in all three groups received intraperitoneal (i.p.) injections. The tumor volume of nude mice was estimated every two days via the eq. V = (L × W^2^)/2, where L is the length, and W is the width of the tumor, and the length and width were measured with vernier calipers. After treatment for 14 days, nude mice of all three groups were sacrificed, and the tumors were removed, weighed, photographed, and fixed in 10% formaldehyde for immunohistochemistry (IHC) and hematoxylin-eosin (H&E) staining. 

### 4.11. Immunohistochemical Analysis

The Universal Two-step kit (PV-9000, Beijing Zhongshan Jinqiao Biotechnology Co., Ltd., Beijing, China) was used for immunohistochemistry, according to the manufacturer’s instructions. Tumor samples from nude mice were embedded in paraffin, then dewaxed, hydrated, repaired by antigen, blocked by endogenous peroxidase, circled around the tumor tissue by immunohistochemistry pen, sealed for 1 h at 37 °C, incubated with primary antibody overnight at 4 °C (The following concentrations of antibodies were used: Ki-67 (Cell Signaling Technology, Boston, USA, 1:800); fumarate hydratase (Proteintech, Wuhan, China, 1:400); and caspase-3 (Proteintech, Wuhan, China, 1:200)), added reaction enhancement solution and enhanced enzyme-labeled goat anti-mouse/rabbit IgG polymer in order, both for 20 min at 37 °C, added freshly prepared DAB, rinsed in tap water and distilled water, incubated with hematoxylin for 2 min, rinsed in tap water for 2 min, returned to blue with lithium carbonate, washed in anhydrous ethanol, dried, sealed, covered. Images were captured using an OLYMPUS BX51 inverted microscope (Tokyo, Japan).

### 4.12. Mass Spectrometry-Cellular Thermal Shift Assay (MS-CETSA)

Cells were collected to prepare cell lysate, washed twice with cold PBS, scraped with the cell scraper, centrifuged, resuspended in PBS containing protease inhibitor and 1.5 mM MgCl_2_ (Sigma, Frankfurter Strasse, Germany), and mechanically broken on ice for 2 min. The concentration of protein was measured by the BCA Protein Quantification Kit (Vazyme, Nanjing, China) and adjusted to 2 mg/mL, and the crude lysate was divided into 2 aliquots: experimental groups treated with compound SIOC-XJC-SF02; control groups treated with DMSO. The concentration of DMSO in control groups was consistent with the experimental groups. Crude lysate was then divided into 16 aliquots for MS-CETSA experiments (8 aliquots treated with compound SIOC-XJC-SF02, 8 aliquots treated with DMSO). Each compound and vehicle were heated with different temperatures in parallel (37.0 °C, 44.0 °C, 46.9 °C, 49.8 °C, 52.9 °C, 55.5 °C, 58.6 °C, and 66.3 °C) for 3 min. The following steps were performed according to the related literature [64,65,66].

### 4.13. Molecular Docking 

The name of the protein was queried by the UniProt database (https://www.uniprot.org/). The pdb format file (PDB code: 5UPP, Resolution: 1.80 Å, RCSB PDB—5UPP: Crystal structure of human fumarate hydratase: https://www.rcsb.org/structure/5UPP) for the protein was downloaded by the PDB database (RCSB PDB: Homepage: https://www.rcsb.org/). The protein was removed from the solvent and organic by PyMOL software (https://pymol.org/2/). The compound SIOC-XJC-SF02 was analyzed and combined with the proteins via Autodock Vina software (https://vina.scripps.edu/) [67]. 

### 4.14. Enzymatic Activity Assay of Fumarate Hydratase

The measuring of mitochondria fumarate hydratase activity was performed using a Fumarate Hydratase Activity Kit (SuZhou Grace Biotechnology Co., Ltd., Suzhou, China) following the manufacturer’s instructions. The optical density was determined at 340 nm after preheating for 30 min.

## 5. Conclusions

This study demonstrated the inhibitory effect of compound SIOC-XJC-SF02 on human HCC cells in vitro and in vivo for the first time and explored the possible mechanisms via MS-CETSA. These results reveal that compound SIOC-XJC-SF02 can exert antitumor effect as a new leading compound. Our findings are also promising for the future design of antitumor activity of a difluorinated alkoxy sulfonyl chloride. The 2-quinolyl-1,3-tropolones were also demonstrated to induce apoptotic cell death of ovarian cancer (OVCAR-3, OVCAR-8) and colon cancer (HCT 116) cell lines and affect ERK signaling [68]. Synthetic compounds could improve the efficiency of antitumor drug research.

## Figures and Tables

**Table 1:** Cytotoxic activity of fenofibrate derivatives against cancer cell lines.

Compound	Cell	IC_50_^a^
SIOC-XJC-SF01	HepG2	>50 μM
SIOC-XJC-SF02	HepG2	10.908 μM
SIOC-XJC-SF03	HepG2	>50 μM
SIOC-XJC-SF04	HepG2	>50 μM
SIOC-XJC-SF05	HepG2	>50 μM
SIOC-XJC-SF06	HepG2	4.182 μM

^a^ IC_50_ values are indicated as the means ± SD (standard error) of at least three independent experiments. The cells were continuously treated with compounds for 48 h.

## Data Availability

The data presented in this study are available on request from the corresponding author. The data are not publicly available due to privacy.

## Supplementary Materials

The following supporting information can be downloaded at: https://www.mdpi.com/article/10.3390/ph16121705/s1, Figure S1. ^1^H NMR spectrum (400 MHz, CDCl_3_) of SIOC-XJC-SF02. Figure S2.^19^F NMR spectrum (376 MHz, CDCl_3_) of SIOC-XJC-SF02. Figure S3. ^13^C NMR spectrum (100 MHz, CDCl_3_) of SIOC-XJC-SF02.
