# Supplementary material for: Identification of a Difluorinated Alkoxy Sulfonyl Chloride as a Novel Antitumor Agent for Hepatocellular Carcinoma through Activating Fumarate Hydratase Activity
Source: Pharmaceuticals (Basel). 2023 Dec 8;16(12):1705. doi: 10.3390/ph16121705 (PMC10748328; doi:10.3390/ph16121705)
Supplement: Supplementary file 1 [file pharmaceuticals-16-01705-s001.zip › pharmaceuticals-2704934-supplementary.pdf]

Supplemental Material

SIOC-XJC-SF02, 2-{4-[4-(2-Fluorosulfonyl-vinyl)-benzoyl]-phenoxy}-2-methyl-propionic acid isopropyl ester (C22H23FO6S), was provided by Shanghai Institute of Organic Chemistry, Chinese Academy of Sciences. The following are the spectral results:

White solid, m. p.: 103-105 ºC, 81% yield. ^{1}H NMR (400 MHz, $CDCl_3$) δ7.87 (d, J = 15.6 Hz, 1H), 7.82 (d, J = 8.1 Hz, 2H), 7.76 (d, J = 8.7 Hz, 2H), 7.70 (d, J = 8.1 Hz, 2H), 7.07 (dd, J = 15.6 Hz, 2.0 Hz, 1H), 6.89 (d, J = 8.7 Hz, 2H), 5.10 (hept , J = 6.1 Hz, 1H), 1.68 (s, 6H), 1.22 (d, J = 6.2 Hz, 6H). ^{19}F NMR (376 MHz, $CDCl_3$) δ62.1 (s, 1F). ^{13}C NMR (100 MHz, $CDCl_3$) δ193.9, 172.8, 159.9, 147.3 (d, J = 2.5 Hz), 141.3, 133.7, 131.9, 130.2, 129.6, 128.7, 119.8 (d, J = 28.4 Hz), 117.2, 79.3, 69.2, 25.2, 21.3. HRMS (ESI): Calculated for C22H24FO6S^{+} [M+H] $^{+}$: 435.1272. Found: 435.1268. Infrared spectrum IR (KBr): 2985, 1730, 1656, 1598, 1408, 1282, 1206, 1102, 975, 930, 873, 764, 628, 557 cm−1 (Figure S1, S2 and S3).

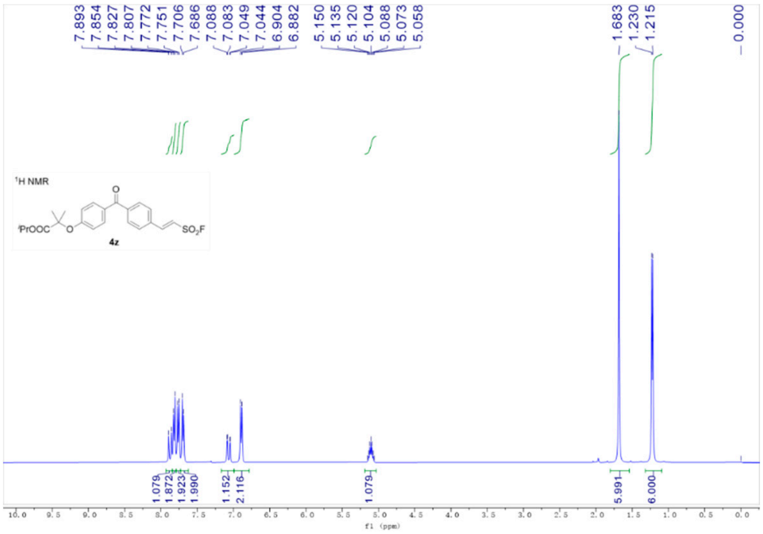

Figure S1. ^{1}H NMR spectrum (400 MHz, $CDCl_3$) of SIOC-XJC-SF02.

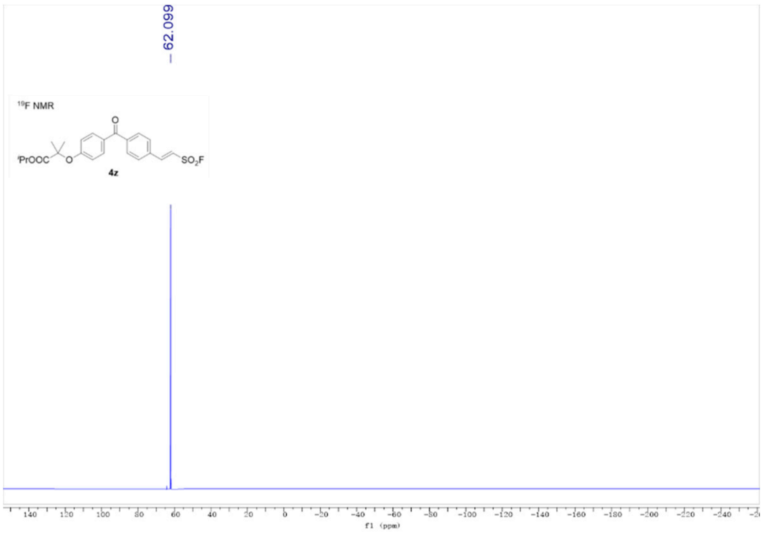

Figure S2. ^{19}F NMR spectrum (376 MHz, $CDCl_3$) of SIOC-XJC-SF02.

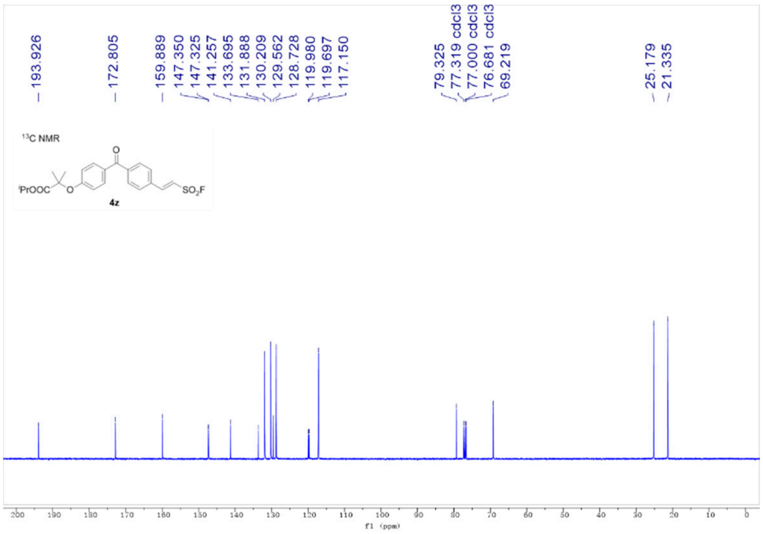

Figure S3. ^{13}C NMR spectrum (100 MHz, $CDCl_3$) of SIOC-XJC-SF02.
